# A novel diagnostic and prognostic approach for unresponsive patients with anthroponotic cutaneous leishmaniasis using artificial neural networks

**DOI:** 10.1371/journal.pone.0250904

**Published:** 2021-05-05

**Authors:** Mehdi Bamorovat, Iraj Sharifi, Esmat Rashedi, Alireza Shafiian, Fatemeh Sharifi, Ahmad Khosravi, Amirhossein Tahmouresi

**Affiliations:** 1 Leishmaniasis Research Center, Kerman University of Medical Sciences, Kerman, Iran; 2 Department of Electrical and Computer Engineering, Graduate University of Advanced Technology, Kerman, Iran; 3 Department of Pathobiology, Faculty of Veterinary Medicine, Shahid Bahonar University of Kerman, Kerman, Iran; 4 Pharmaceutics Research Center, Institute of Neuropharmacology, Kerman University of Medical Sciences, Kerman, Iran; Ohio State University, UNITED STATES

## Abstract

Cutaneous leishmaniasis (CL) imposes a major health burden throughout the tropical and subtropical regions of the globe. Unresponsive cases are common phenomena occurred upon exposure to the standard drugs. Therefore, rapid detection, prognosis and classification of the disease are crucial for selecting the proper treatment modality. Using machine learning (ML) techniques, this study aimed to detect unresponsive cases of ACL, caused by *Leishmania tropica*, which will consequently be used for a more effective treatment modality. This study was conducted as a case-control setting. Patients were selected in a major ACL focus from both unresponsive and responsive cases. Nine unique and relevant features of patients with ACL were selected. To categorize the patients, different classifier models such as k-nearest neighbors (KNN), support vector machines (SVM), multilayer perceptron (MLP), learning vector quantization (LVQ) and multipass LVQ were applied and compared for this supervised learning task. Comparison of the receiver operating characteristic graphs (ROC) and confusion plots for the above models represented that MLP was a fairly accurate prediction model to solve this problem. The overall accuracy in terms of sensitivity, specificity and area under ROC curve (AUC) of MLP classifier were 87.8%, 90.3%, 86% and 0.88%, respectively. Moreover, the duration of the skin lesion was the most influential feature in MLP classifier, while gender was the least. The present investigation demonstrated that MLP model could be utilized for rapid detection, accurate prognosis and effective treatment of unresponsive patients with ACL. The results showed that the major feature affecting the responsiveness to treatments is the duration of the lesion. This novel approach is unique and can be beneficial in developing diagnostic, prophylactic and therapeutic measures against the disease. This attempt could be a preliminary step towards the expansion of ML application in future directions.

## Introduction

Recently, there have been advancements and achievements in various fields of science, particularly medicine. The overlap among different fields of science has also made a great contribution to this progress. Medical artificial intelligence (AI) is a good example of the intersection of computer and medical sciences. An important application of AI is machine learning (ML) algorithms and translation of its diagnostic capability into various fields of medicine and biology [1–6]. Use of ML and its various methods in classification and pattern recognition in medical diagnosis improves the accuracy, speed and efficiency of the conclusion procedure. Using different methods and algorithms, the learning process of ML requires observations and labels for classification and pattern recognition, which are considered as supervised learning, to identify patterns among the data [1]. Previous researches have used various methods such as support vector machine (SVM), decision tree and different artificial neural network (ANN) structures. Since the diagnosis process is regarded as a classification problem in ML, different methods such as ANN structures can be used for this purpose. Classifiers have been used to identify the patterns among data in different medical diagnosis and prognosis approaches [7]. In previous studies, ANN structures were the most commonly used method in the detection and diagnosis process in various medical fields [8]. They have also been surveyed in classification processes for medical decision-making from ML perspectives [9].

Leishmaniasis is one of the main neglected and emergent tropical diseases, which comprises of a complex of different clinical manifestations caused by more than 20 species of the genus *Leishmania*. The disease consists of three typical and clinical disorders including visceral leishmaniasis (VL), mucocutaneous leishmaniasis (MCL) and cutaneous leishmaniasis (CL) [10–12]. The prevalence of leishmaniasis is dynamic and the disease is reported to (re-)emerge and expand its traditional territories to new foci in many countries [13, 14]. According to the most recent estimate, 26,000 to 65,000 deaths occur annually worldwide and more than one billion inhabitants in over 100 countries and territories are at risk of leishmaniasis (431, 850, 220 million people for CL and 616, 220, 219 million people for VL). In 2017, more than 95% of the new CL cases occurred in 7 countries: Afghanistan, Brazil, Colombia, Algeria, Iraq, Iran (Islamic Republic of), and the Syrian Arab Republic [12].

With high outbreak rates, anthroponotic CL (ACL) due to *Leishmania tropica* is endemic in several parts of Iran and many countries as well [15–18]. The disease occurs in humans anthroponotically by the bite of female sand flies [19]. Since humans are the main reservoir host, the control strategy should be centered on early diagnosis and effective treatment for the disease. For more than seven decades, antimonial drugs such as meglumine antimoniate (MA, Glucantime) and sodium stibogluconate (Pentostam) have been the gold standard treatment for CL worldwide. Ever since, MA resistant *L*. *tropica* isolates have emerged from unresponsive cases. Unresponsiveness to treatment is a multifactorial condition which could be associated with social and demographical factors, duration and type of the disease, type of the drugs and host’s immune response [20–24]. Recently, challenges in the chemotherapy of ACL has generated considerable resistance and resulted in treatment failure and relapse cases (refractory cases) [1, 25]. Based on the recent studies in southeastern Iran, several contributory features such as age, duration of the lesion, history of chronic diseases and housing condition have played a significant role in the induction of the unresponsive forms [24, 26]. Therefore, appropriate diagnosis and classification of leishmaniasis are very important for proper prognosis and treatment of the patients.

Extensive studies have been conducted for detection and classification of the diseases worldwide. Tanvi Gupta et al [27] accomplished a research using several ML methods to classify 200 patients as normal and abnormal cases. Accordingly, 12 extracted features from MRI images were included. The linear SVM was the best classifier with 88% accuracy. In another study, a classifier model was considered for cancer diagnosis based on the gene signatures using ANNs. The neural network train process was performed with small round blue-cell data. These cancers were divided into 4 distinct classes and the neural network was able to classify them correctly [28]. Alzubi et al [29] used a reinforced neural network to diagnose lung cancer disease more accurately. The results showed that this new approach improved and increased the accuracy of prediction and reduced the duration of diagnosis procedure, compared to the conventional methods of diagnosis and prognosis. According to the previous studies, different machine learning methods help the process of diagnosis by developing classifier models through different algorithms and methods to take a further step in medical sciences. Correct detection of the disease and precise classification of unresponsive and responsive forms in ACL patients are beneficial for selecting the proper treatment modality, predicting the likely or expected development of the disease and helping health care providers to be more mindful in detecting such cases carefully.

The present study was aimed to explore a novel approach by implementing ML methods, notably ANN, for rapid diagnosis, prognosis and classification of unresponsive vs. responsive forms in patients with ACL in the main focus in southeastern Iran.

## Materials and methods

### Study design

A case-control study was conducted between April 2015 and May 2019 in high-risk zones of ACL in Kerman district, in the southeast of Iran, at Dadbin CL clinic, affiliated with the Leishmaniasis Research Center, School of Medicine, Kerman University of Medical Sciences. This study was performed in two groups; in fact, two groups had two different consequences in terms of treatment outcome. One group responded to treatment (control) and the other group (case) did not. This is while the situation in both treatment groups was similar to the drug (MA) and also matching was performed in terms of sex, age, education and zone of sampling. Therefore, one group was considered as a control and the other group as a case,

### Ethical consideration

The ethical consideration procedure of this study was specifically approved by the joint Ethical Committees of Kerman University of Medical Sciences and Kerman Leishmaniasis Research Center (ethic no. IR. KMU. REC.1398.428, contract no. 98000673). At first, several face-to-face meetings and interviews with the patients and community authorities were arranged. The aims of the study, procedures, and potential gains were explained. CL patients participated voluntarily in the study and written informed consent was obtained from all of them. All demographic, clinical, and environmental data were kept strictly confidential. Furthermore, “on behalf" of all the children, parents/guardians completed the written informed consent. The data source originated from demographical and clinical histories of unresponsive and responsive patients, which were recorded in the electronic software and back up registration book in the CL clinic.

### Study site

Kerman as the largest province of Iran and a major focus for ACL is located in the southeast Iran. The province falls into the arid and semi-arid regions and suffers from the scarcity of water, conditions like much of the Iranian plateau. The regular annual rainfall is low and maximum precipitation happens in winter, with the average annual rainfall of 142 mm in Kerman city. Leishmaniasis, with an old history, is endemic in the province. Two major ACL foci including Kerman and Bam districts exist in the province [30].

### Questionnaire form and selection of the features

The questionnaires of the study were designed according to the most relevant CL indicators. In fact, the features and queries of the questionnaire were chosen according to the identification of significant risk factors for unresponsiveness to treatment in patients with ACL in the previous studies [24, 26, 31, 32] and importance of health issues related to demographical, clinical and environmental characteristics of CL from the expert’s points of view. The input vector for each record included nine features, as the most relevant disease indicators, namely interior housing condition, age, sex, education, duration of the lesion, number of the lesion, location of the lesion, treatment course and history of underlying chronic diseases. Participant patients or their guardians were informed of the essential details about the study. Then, the interview processes and medical examinations were carried out. Throughout the work, one made sure that the participant or their guardians well understood the queries. Data were collected by house-to-house visits using structured questionnaires. Some patients whose names, demographics, and clinical characteristics were recorded in the registry system but were absent at the house during the follow-up assessments, unable to record environmental data and therefore, were excluded from the study. Moreover, the interviewers conducted direct visit and careful observation of the housing and environmental conditions and data were recorded with the consent of participants. The data were correctly documented by the interviewers recording demographic, clinical and environmental characteristics.

### Case-definition

**Fig 1:**
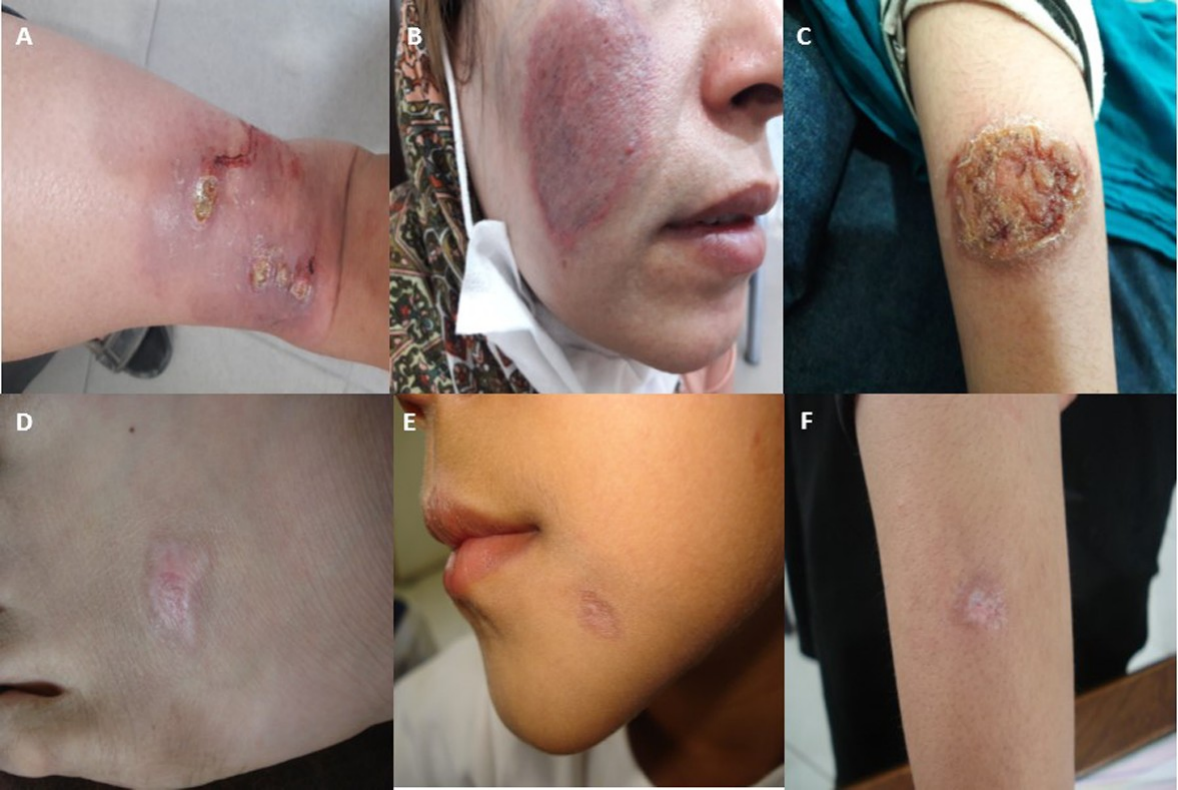
A, B and C; representative images of unresponsive cases, D, E and F; representative images of responsive cases with anthroponotic cutaneous leishmaniasis due to *Leishmania tropica* from a major focus, Kerman, southeast of Iran.

Patients were selected from a major ACL focus from unresponsive and responsive forms as case and control groups, respectively. The unresponsive patient is one who has not been cured and remained with an active lesion, despite receiving three courses of intra-lesional MA (20mg/kg/weekly) along with biweekly liquid nitrogen cryotherapy for 12 weeks or systemic MA alone (20 mg/kg/daily for 3 weeks) and at least one year has passed since the appearance of his/her lesion (Fig 1A–1C). The responsive patient is one whose lesion has healed by one treatment cycle with intramuscular administration of MA alone or intra-lesional MA together with biweekly cryotherapy without CL recurrence after 6 months of the follow-up assessment (Fig 1D–1F).

Overall, 172 ACL patients consisting of 72 (41.86%) unresponsive and 100 (58.14%) responsive cases (Table 1) were analyzed in the study. This sample size (n = 72) was selected according to the rate of unresponsive patients in this region (10 to 12%, based on our knowledge and experience of the ACL cases referred to the registry center in Kerman and according to the previous studies), which is significantly lower than the control group (responsive patients). Data of features in unresponsive and responsive cases (treatment outcome) with ACL showed in S1 Dataset.

**Table 1:** Baseline features of unresponsive and responsive patients with anthroponotic cutaneous leishmaniasis.

Features	Categories of features	Number of unresponsive cases (%)	Number of responsive cases (%)
Interior housing condition	Suitable^a^	38 (52.78)	69 (69)
	Unsuitable^b^	34 (47.22)	31 (31)
Age (year)	≤ 7	19 (26.39)	25 (25)
	8–15	18 (25)	30 (30)
	16–30	13 (18.05)	25 (25)
	31–50	13 (18.05)	14 (14)
	≥ 50	9 (12.5)	6 (6)
Sex	Female	30 (41.67)	53 (53)
	Male	42 (58.33)	47 (47)
Education	Illiterate	31 (43.05)	26 (26)
	Primary and secondary	31 (43.05)	46 (46)
	High school and university	10 (13.89)	28 (28)
Duration of lesion (month)	≤4	7 (9.72)	53 (53)
	5–12	47 (65.28)	45 (45)
	≥13	18 (25)	2 (2)
Number of lesions	≤2	65 (90.28)	83 (83)
	≥3	7 (9.72)	17 (17)
Location of lesion	Hand	25 (34.72)	51 (51)
	Face	38 (52.78)	31 (31)
	Other	9 (12.5)	18 (18)
Treatment course	Incomplete^c^	16 (22.22)	24 (24)
	Complete^d^	56 (77.78)	76 (76)
History of chronic diseases	Yes	22 (30.55)	6 (6)
	No	50 (69.45)	94 (94)
Total		72 (41.86)	100 (58.14)

a: suitable: denotes the patients who live in an appropriate housing condition in terms of building and interior housing, with no cracks and crevices,

b: unsuitable: denotes the patients who live in inappropriate housing conditions in terms of building and interior housing,

c: incomplete treatment: the patients who did not receive a complete course of intramuscular (IM) or intralesional (IL) treatment along with cryotherapy,

d: completed treatment: the patients who received a full course of treatment schedule.

### Machine learning in bioinformatics

ML is a field of science and technology in which computers make different models for varying purposes concerning the inference of data patterns. Training data are used to train algorithms or ML models to be used for diagnosis, prediction and decision-making classification. ML is widely used, especially where common algorithms do not have the proper efficiency. Data mining or pattern inference process in data sets is used in ML. Subjects in ML are divided into three general categories of supervised learning, semi-supervised learning and unsupervised learning. Due to the structure of the problem, their methods and algorithms are applicable [33].

Classification and regression algorithms are supervised learning. In classification problems, the input labels are related to the number of each class, while the outputs in the regression are continuous numbers in the specified range. In unsupervised learning, a mathematical model is constructed for a set of data whose outputs or labels are not specified. The purpose of this type of training is to find the pattern and structure of the input data in order to be divided into groups, which are called clusters. In semi-supervised learning, some of the input data are labeled and the mathematical model of incomplete training data is developed [34].

ML is closely linked to optimization and statistics. Parameters of the models built in ML with different optimization tools are chosen in a way that the model have the closest prediction to the training data labels. There are many grey areas where ML and statistics collide. The statistical methods rely on probability functions to derive their prediction methods, whereas ML are not restricted to probabilities and can make use of any mathematical function to connect outputs to inputs as long as usable predictions can be obtained [35, 36]. According to the classification description in ML, which is one of the supervised learning methods used in problems where specific data labels are determined, class numbers are given and the classifier algorithms find this diagnostic pattern.

### Classifiers in machine learning

A classifier is an algorithm for sorting data into their class labels. Classification is introduced as a supervised learning problem in ML. The algorithm will provide the training data whose output is clear to train the pattern among the data. In unsupervised learning, issues such as clustering are introduced. In fact, a classifier model is defined by a mathematical function, which maps entries to a desired class. Input feature vectors are known as independent variables and category or output variables are known as dependent variables. The feature vector is identified and selected according to the problem type [37].

Various algorithms and methods have been proposed to classify and construct a model for this purpose. The most common methods in ML are various ANN structures, decision trees (DT), k-nearest neighbors (KNN) and SVM structures which have previously been used in many diagnostic methodologies in medicine [7–9, 28, 29, 38, 39].

**Fig 2:**
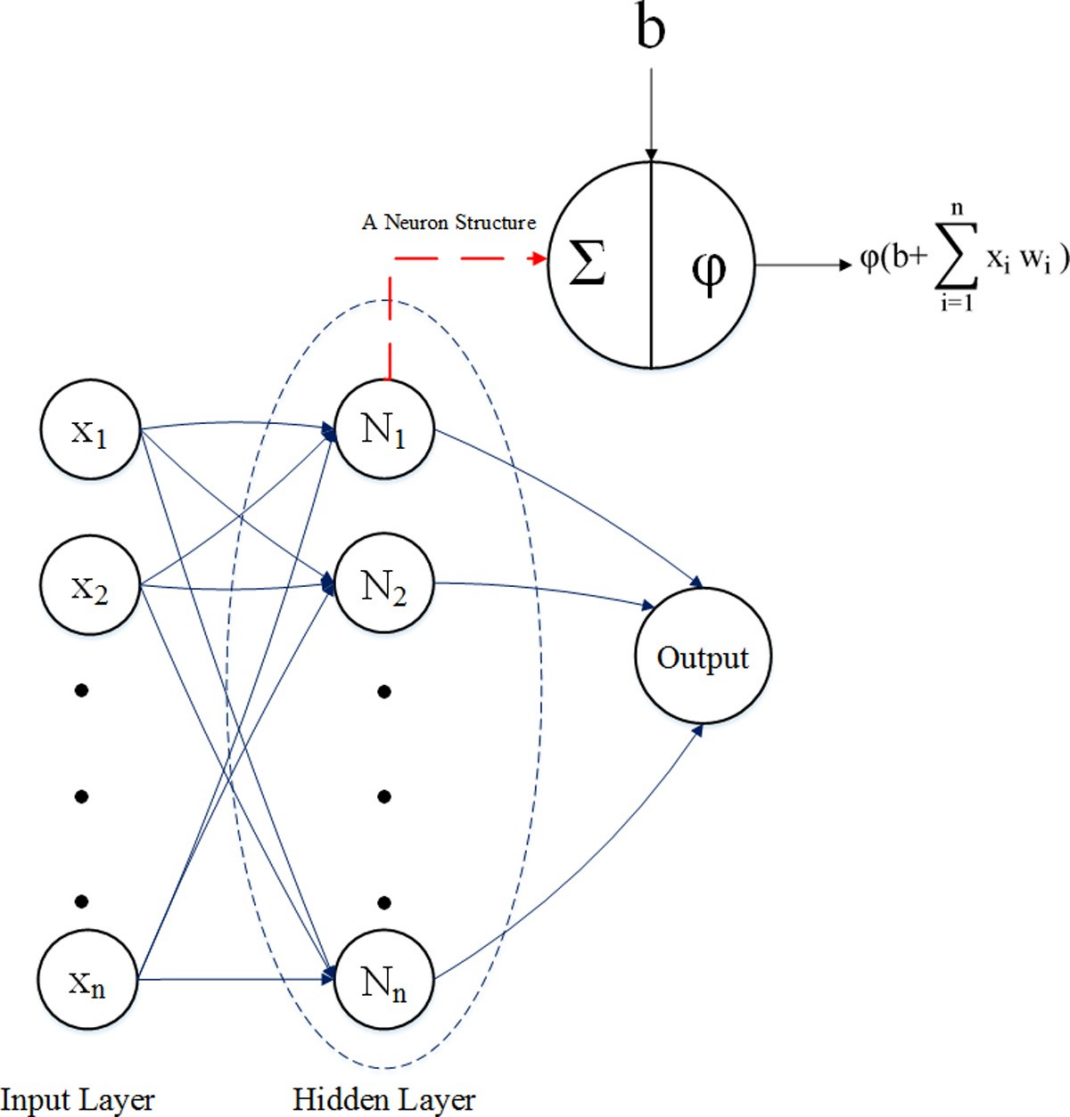
Structure of the multilayer perceptron with one hidden layer.

This study was built on the multilayer perceptron (MLP) structure of the feed forward ANN class alongside two versions of the learning vector quantization (LVQ) method, which is a class of ANNs. Moreover, support vector machine (SVM) and k-nearest neighbors (KNN) were utilized for better comparison in this classification task. MLP consists of at least three layers including input layer, hidden layer and the output layer. There are several neurons in each layer where an activation function has been used in each neuron, which is the most important part of MLP. Fig 2 displays a framework of MLP.

Input vector containing specified properties for each record is entered into the neural network with the specified number of the hidden layers and neurons of each layer. Then, the weighted inputs Xi W^T^i and their sum with bias ‘b’ are passed through the non-linear functions (φ) called activation functions that are presented in the next layer. Various gradient algorithms perform the learning process, which actually optimizes the weights and biases of the network. Additionally, the most suitable network structure (number of layers and neurons per layer) should optimally be determined with the least error [40].

LVQ, also known as a neural network, is used for input vector classification problems, which is based on the winner, which takes all approaches. The LVQ consists of two main steps. In the first step, cluster centers are defined by the operation of an unsupervised learning algorithm. Then, in the next step, based on the classes information, the cluster centers will be improved to minimize the number of misclassified observations [38].

In the competitive layer, the Euclidean distance between the input vector and the Wi is calculated. Then, the centers are updated according to the learning degree. Different versions of LVQ, including LVQ1, LVQ2.1 and multipass LVQ are implemented [41]. In LVQ1, the best matching unit is selected and moved to or away from the data vector. In LVQ2.1, two best matching units are selected and the update operation occurs if one belongs to the description class and the other does not. However, multipass LVQ is the recommended usage of the algorithm [42], that pass is made on the model using LVQ1, and then fine-tuning the next model with LVQ2.1.

Support vector machine known as SVM is used in a wide range of ML problems especially classification issues with set of training examples each labeled with the classes. SVMs are efficiently performed in both linear and non-linear classification space by utilizing kernel trick, essentially mapping inputs into high-dimensional attribute space [43, 44].

Additionally, in classification and pattern recognition, the k-nearest neighbors (KNN) is one of the most significant and simple classification methods, which is the first choice when the distribution of the data is unknown [45].

### Proposed method

**Fig 3:**
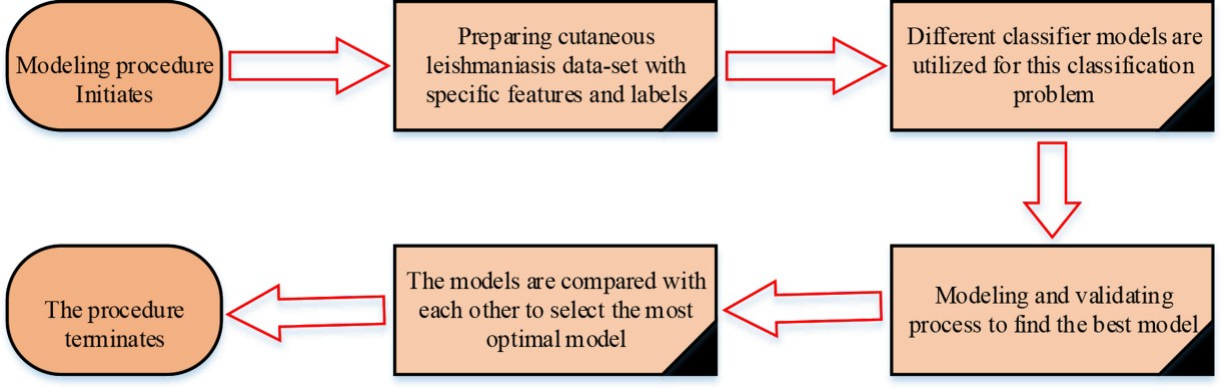
Overall procedures for modeling.

In this study, several approaches were investigated in order to classify CL patients with unresponsive and responsive labels. Therefore, the output represented two classes including unresponsive and responsive cases. The input vector for each record included 9 features as the most relevant Indicators of the disease, which were designated as interior housing condition, age, sex, education, duration of the lesion, number of the lesion, location of the lesion, treatment course and history of chronic diseases. These selected attributes were described for conditions of 172 patients. Based on this issue, which can be modeled as a classification model in ML, different classification models were implemented. Thus, MLP, SVM, KNN and two variants implementation of LVQ (LVQ1 and multipass LVQ) were utilized. These classifier models were compared with each other in terms of accuracy. In order to prevent overfitting problem and to obtain a more reliable model, all of the 172 cases were divided into 3 types of data for training, test and 15% validation of the structured networks. The appropriate learning parameters were applied for models to find the most optimal classifier. After tuning the models by learning procedure, the classifiers can be applied. Moreover, hyperbolic tangent (TANSIG) is utilized as an activation function in MLP structure. The LVQ learning factor is regulated to 0.01 and radial basis (RBF) function is operated for SVM kernel function. In KNN algorithm, k value is considered as 5. Furthermore, all mentioned parameters are applied to obtain the suitable and efficient models in this issue. The overall process in order to achieve this purpose is presented in Fig 3.

## Results

**Fig 4:**
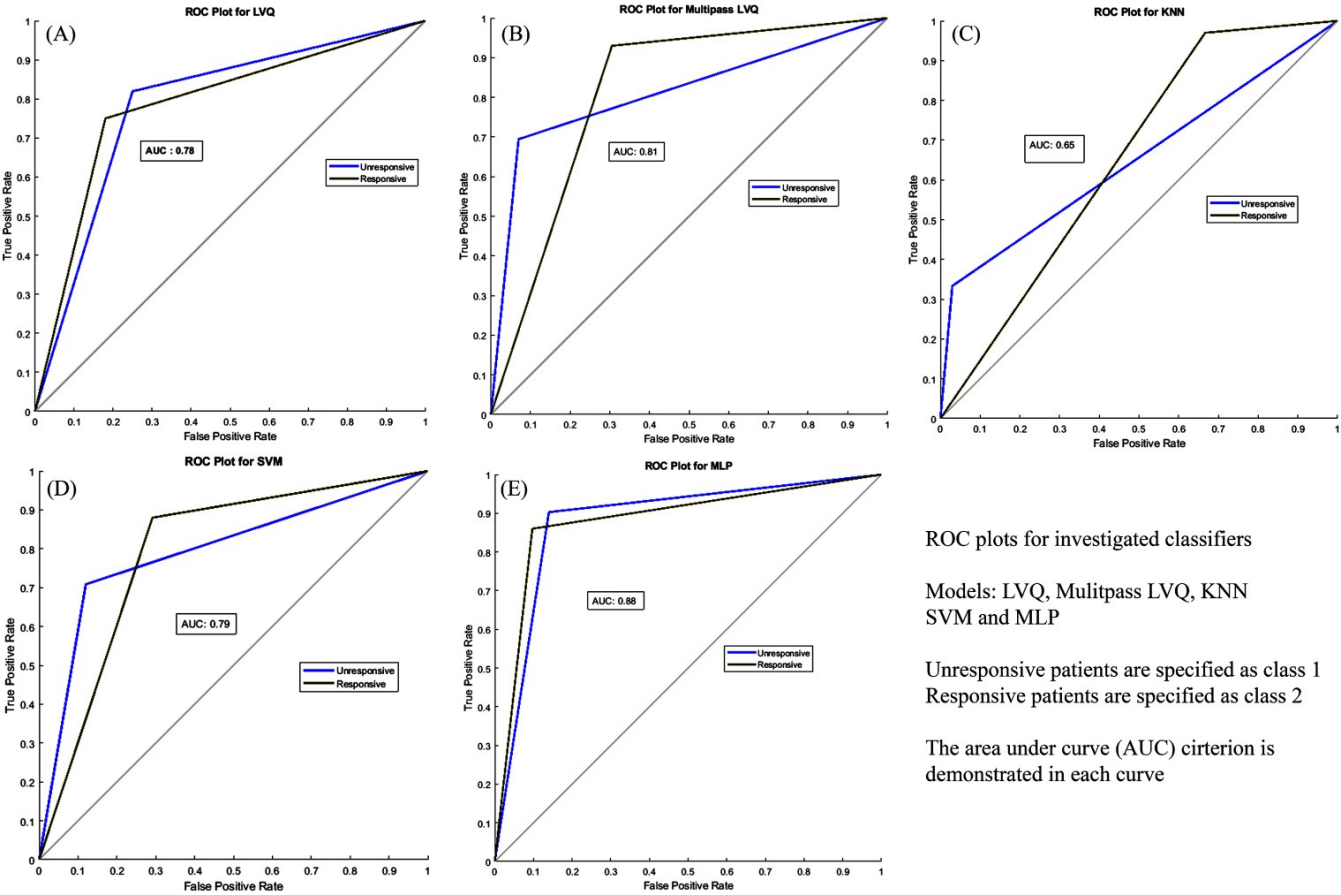
A, Learning vector quantization neural network ROC plot was prepared for unresponsive and responsive classes; B, Multipass learning vector quantization neural network ROC plot was prepared for unresponsive and responsive classes; C, Multilayer perceptron neural network ROC plot of unresponsive and responsive classes; D, Support vector machine ROC plot of unresponsive and responsive classes; E, K-nearest neighbors ROC plot of unresponsive and responsive classes.

In the present study, the process of modeling was carried out with five structures of classifiers. Moreover, different graphs are presented for the models. These modeling processes were implemented in MATLAB. The results demonstrated that the most suitable classifier for our classification problem was MLP classifier; therefore, the receiver operating characteristic graph (ROC), which illustrated the diagnostic ability of a classifier model, was provided. This plot had two various threshold settings that were named as the true positive rate (TPR) against the false positive rate (FPR) [46]. Furthermore, true positive rate is known as the sensitivity or probability of detection in ML and false-positive rate is defined as the probability of false alarm (or 1-specificity). In further details, sensitivity measures the proportion of actual positives that are correctly identified, while specificity measures the proportion of actual negative cases that were correctly identified. ROC analysis was related to the diagnostic decision-making; the closer a result was to the upper left corner, the more efficient the classifier model was. LVQ, multipass LVQ, MLP, SVM and KNN ROC plots with the area under curve parameter (AUC) are presented in Fig 4.

**Fig 5:**
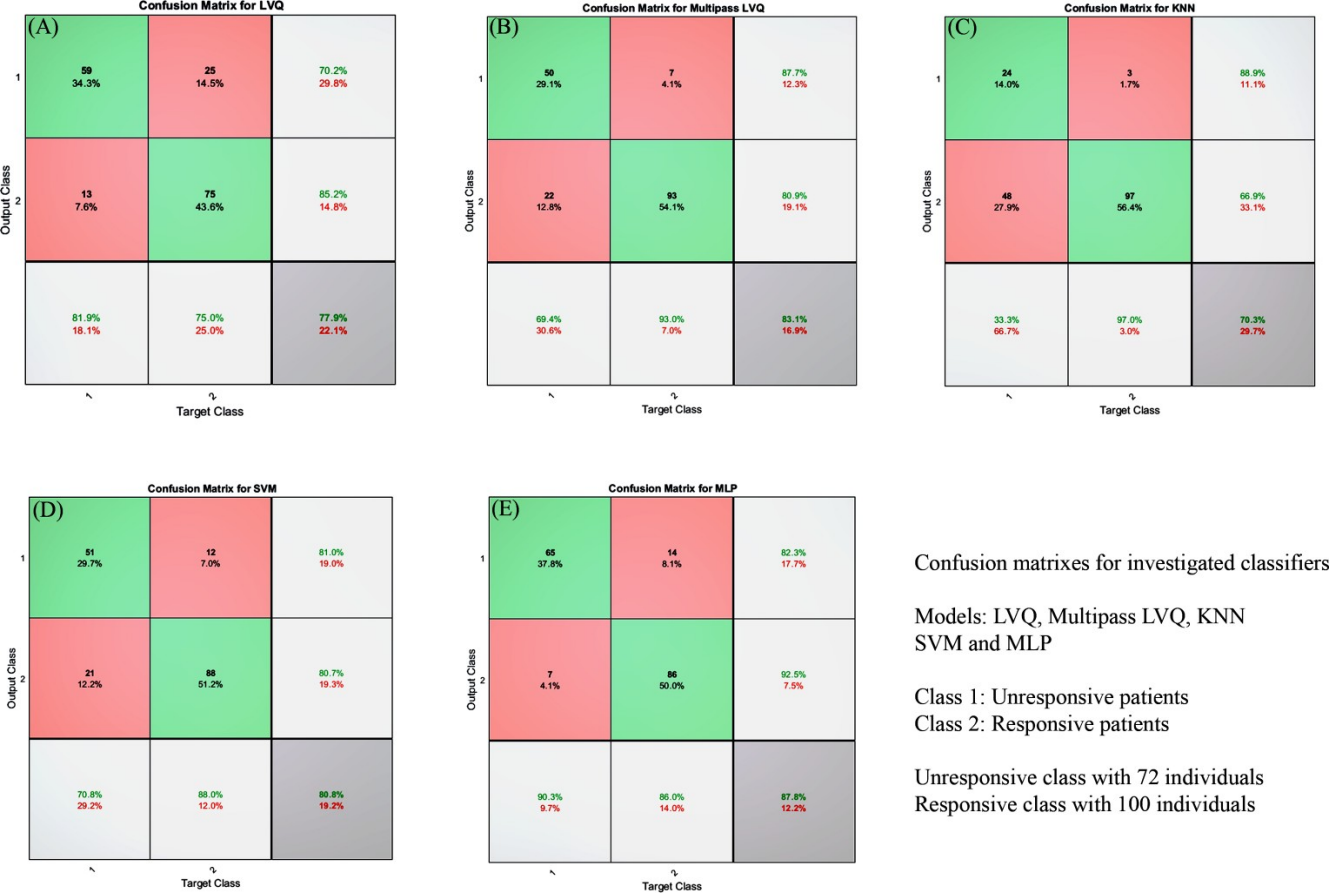
A, Confusion matrix for learning vector quantization neural network representing the performance of the model; B, Confusion matrix for multipass learning vector quantization neural network representing the performance of the model; C, Confusion matrix for multilayer perceptron neural network representing the performance of the model; D, Confusion matrix for support vector machine representing the performance of the model; E, Confusion matrix for k-nearest neighbors representing the performance of the model.

In order to have better analyses, confusion matrix or error matrix plots, which illustrate the performance of a classification model, were presented [27, 46]. These plots are the summary of the prediction results on the issue. Confusion matrix graphs are demonstrated in Fig 5. To have a better insight into the performance of the classifier, Table 2 is prepared for comparing the models.

**Table 2:** Classification results for the whole data.

Classifier	Sensitivity	Specificity	Accuracy	AUC
MLP^a^	90.3%	86%	87.8%	0.88
Multipass LVQ^b^	69.4%	93%	83.1%	0.81
LVQ^c^	81.9%	75%	78%	0.78
SVM^d^	70.8%	88%	80.8	0.79
KNN^e^	33.3%	97%	70.3%	0.65

a: multilayer perceptron,

b: multipass learning vector quantization,

c: learning vector quantization,

d: support vector machine,

e: k nearest neighbors.

Furthermore, three main criteria of accuracy, sensitivity and specificity, which are utilized in medical classification systems, were identified [8]. Moreover, Eqs 1, 2 and 3 calculate the accuracy, sensitivity and specificity, respectively.

$$Accuracy=\frac{\text{Correctly}\;\text{classified}\;\text{pateints}}{\text{Total}\;\text{patients}}\times 100$$ (1)

$$Sensitivity=\frac{\text{Correctly}\;\text{indentified}\;\text{unresponsive}\;\text{pateints}}{\text{Total}\;\text{unresponsive}\;\text{patients}}\times 100$$ (2)

$$Specificity=\frac{\text{Correctly}\;\text{indentified}\;\text{responsive}\;\text{patients}}{\text{Total}\;\text{responsive}\;\text{patients}}\times 100$$ (3)

Moreover, the best MLP structure was utilized in this study in order to determine the effectiveness of each feature. Hence, when one of the features was removed, the MLP was trained with the remaining eight features and the accuracy of the total data was reported. This process was repeated for each of the features and the results are presented in Table 3. Based on these findings, duration of the lesion, as the fifth feature (F5) in the data set, was the most influential feature because its removal resulted in the least rate of accuracy (66.9%), while sex was the feature with the least efficiency in the test.

**Table 3:** Removing features in order to determine the effectiveness of each one.

	F^a^1	F2	F3	F4	F5	F6	F7	F8	F9
Removing Feature	Interior housing condition	Age	Sex	Education	Duration of lesion	Number of lesion	Location of lesion	Treatment course	History of chronic diseases
**The classification accuracy (%)**	78.5	82	84.3	76.2	66.9	83	83.1	80.2	74

^a^ Feature,

^b^ The classification accuracy of total data (%) with the remaining features.

## Discussion

Global health care costs are estimated to reach $8.7 trillion by 2020, driven by aging people growing in size, aging and disease complexity, progresses made in medical treatments, increasing labor costs and market development of the health care system [47]. CL is one of the most important health problems in the world, particularly in Iran [17, 48]. Previous surveys have shown that CL in Kerman district was frequently of the anthroponotic type (ACL) caused by *L*. *tropica* [24, 26, 49]. Unresponsiveness to treatment is an important and recognized problematic feature in response to the therapeutic regimen not only seen in parasitic diseases such as leishmaniasis but also in bacterial and fungal diseases [24, 50, 51]. Rapid detection and accurate prognosis are prerequisites for selecting the proper treatment modality and critical prophylactic approaches for the prevention of unresponsive forms of ACL. Imperative advances have recently been made regarding leishmaniasis, with a focus on its diagnosis, clinical management and appropriate therapy associated with unresponsiveness. Development in diagnostic methods with high levels of sensitivity and specificity are fundamental approaches for the control of the disease. The basic impression of conducting a diagnostic test is to reduce our suspicion and improve the diagnosis that a patient has a specific disease, to the extent that we can make correct decisions. In the present study, we have tried to explain the rationale behind an ANN model and its scientific application in the practical management and selection of the appropriate treatment modality for prevention of unresponsiveness in patients and eventually to control the disease.

As demonstrated in the results, the MLP classifier was recognized as the most optimal prediction model in terms of accuracy (both sensitivity and specificity) of the test. This model of classification can be applied in our investigation with responsive and unresponsive patient classes as an output. Furthermore, patients were categorized in these two classes by 9 attributes as mentioned above. Initially, three ANN architectures were constructed, and then, the best model relevant to its accuracy was introduced as a classifier prototype. Hence, the data set was prepared with 9 key features of two named classes each.

Since our main problem was a classification in machine learning, the issue was presented with MLP, LVQ, multipass LVQ, SVM and KNN classifiers. These methods are implemented in supervised learning problems. This approach could be useful in making an accurate and rapid diagnosis and prognosis in order to detect potential ACL patients who are unresponsive or responsive to the treatment. It seems that awareness of the possibility of development of the unresponsive patients with ACL could be very helpful to select the appropriate treatment modality. Since the reliability of the model and its efficiency rely on the appropriate feature selection, some features may increase the accuracy of the model, which might not be considered in this investigation. Because of the importance of selecting feature procedure, unsuitable selection may become a limitation on model certainty and generalizability.

Several other forms of artificial intelligence have been carried out in medicine with different levels of success. Moreover, numerous studies revealed that ANN and statistical methods can be used in prediction and classification problems in medicine [47, 52, 53]. Due to frequent use of medical information systems, nowadays, ANN is known to be valuable and suitable for implementation in biomedical areas in diagnosis and monitoring of the diseases [54]. Ultimately, a neural network analysis possesses conceivably a principal capacity over the conventional methods when the significance of a given prognostic variable is expressed as a complicated unknown function [55].

For the purposes of analysis, each variable can be considered as a single dimension in a multidimensional space. Some classic analysis techniques are particularly suitable for low-dimensional complexity data analysis and for the analysis of data with a linear separation. These data can be expressed in two dimensions by drawing a straight line between the two populations in a Cartesian graph [48]. On the other hand, the traditional techniques have no capability when a complicated multidimensional non-linear function governs the relationship between the variables [49]. Therefore, in these situations, a neural network can make a more correct analysis [56]. As described, ANN provided much greater predictive accuracy for the possibility of development of unresponsiveness in ACL patients in this study; moreover, this approach has no significant adverse event. This finding resulted in the selection of proper drugs and adherence to treatment in the patients who have the potential of developing unresponsive form.

In a study by Suberi et al., a classifier using ANN and MLP structure was presented. In this classification, posterior fossa slices in CT brain images were sorted for further studies in the diagnosis of stroke. Eleven features were considered for this modeling and this classifier had satisfying accuracy [39]. In another study, the neural network model was used based on the LVQ algorithm to classify electrocardiogram signals. The MIT-BIH arrhythmia database was evaluated with 15 classes, the approach provided good results because of using the whole database [38]. In other studies, researchers showed that using the clinical features including the physical signs, biochemical markers and serological indices could predict the parasite (*Leishmania infantum*) load in the lymph nodes of dogs by radial basis of artificial neural network (RB-ANN) method [57]. Another study showed that ANNs might be applied as a diagnostic and predictive method for tuberculosis (TB) and suggested that they could be supportive in developing the role of computer technologies in rapid diagnosis for the management and control of TB [58]. Moreover, by the neural networks method, diabetic retinopathy and categorizing cancers of skin have been diagnosed [59, 60].

In addition, ANNs have successfully been used in computational biology and bioinformatics in inferring target gene expression, predicting RNA-binding protein sites and biomarkers [61–63]. In a study for prediction of the incidence of malaria, researchers used a new back-propagation neural network (BPNN) classification model. They showed that BPNN model was well suited for deciphering the risk of acquiring malaria as well as other infectious diseases [64]. Currently, there are an increasing number of studies on the use of machine learning methods for diagnosis of malaria. There are techniques of analysis available for the detection and staging of red blood cells (RBCs) infected by the parasite of malaria. Several types of machine learning algorithms as RBC classifiers are used for the classification of RBCs [65–68].

The ANNs are also appropriate for complex diseases and are beneficial with a greater degree of correlation in multivariable classification problems. Disease diagnosis is a good example of such complex problems of classification. By correct application of ANN in this area, this dependency can be extended to obtain the interdependence of signs and proper diagnosis [69–71]. In this study, we attempted to build an adaptive system such as ANN to predict unresponsive ACL forms based on the consequence of the mentioned features. The data also revealed the identification of some suitable features that affect the classification of the responsive and unresponsive patients. Furthermore, the results detected a proper model of MLP that can be used to predict and prognose the unresponsive ACL form based on available features. The correct selection of features in the ANN models was the most important principle to achieve acceptable classification outcomes. The results of our study clearly indicated that the features were correctly selected with relatively high sensitivity and specificity. Our results specifically demonstrated that the duration of the lesion was the most effective feature influencing the patient’s responsiveness to the treatment. In this study, the most influential features that had important roles in the ML models were including duration of the lesion, history of chronic diseases and education level.

A study showed among the patients with CL who had started their treatment regimen after a long time or with incomplete treatment, had greater odds of unresponsiveness than those who started their treatment schedule after a short onset of the disease [24]. Therefore, in the former group, the treatment process was more problematical. Incomplete or delay in treatment is sometimes due to increased drug resistance and type of immune response of the *Leishmania* [72, 73].

The pathogenesis of the increased incidence of infection in patients with a background of chronic diseases such as diabetes and opium addiction is affected by the innate immune response. Therefore, the increased incidence of infections could be due to deficient immunity [16, 50, 74]. In a study, CL patients with a history of chronic diseases developed unresponsive forms more significantly than those without a history of chronic diseases.

The CL patients with a low level of education develop infections more frequently than those with higher education due to lack of knowledge about the disease. It appears that because of the prolonged duration of the disease, poor adherence and untimely treatment course, the possibility of developing unresponsiveness significantly increases in these individuals.

Moreover, in this approach the findings displayed a significant decrease in the duration of diagnostic procedures compared to the conventional diagnostic methods. Whilst, in conventional diagnostic methods, after treatment courses and the consequent treatment failure, patients will be identified as unresponsive cases. Influenced by advancements in this field, decision-makers take advantage of ANN’s hybrid models to adapt approaches to a particular problem. ANN-based solutions used at various decision-making levels suggest the promise of their use in contexts involving complex, limited and/or unstructured information [47].

## Conclusion

This study presented a new diagnostic and prognostic method in the classification of ACL, including both responsive and unresponsive patients, which help physicians to select the proper treatment modality. ANN, particularly MLP, could be an important tool for rapid detection and accurate prognosis of ACL unresponsiveness. The findings could be a warning to select the proper drugs and result in good adherence to therapy in patients who have the potential of developing unresponsive forms. This novel approach in the detection of ACL forms can help develop prophylactic and therapeutic approaches to control the disease. The present study was designed to guide investigators to introduce and advance the potential and beneficial aspects of ML in prompt and precise identification of ACL disease in clinical settings. This attempt could be a primary step towards the expansion of ML application in future directions.

## Supporting information

S1 Checklist STARD checklist. (PDF)

S1 Dataset Data of features in unresponsive and responsive cases (treatment outcome). (XLS)
